# Reduced Ejection Fraction in Elite Endurance Athletes: Clinical and Genetic Overlap With Dilated Cardiomyopathy

**DOI:** 10.1161/CIRCULATIONAHA.122.063777

**Published:** 2023-12-18

**Authors:** Guido Claessen, Ruben De Bosscher, Kristel Janssens, Paul Young, Christophe Dausin, Mathias Claeys, Piet Claus, Kaatje Goetschalckx, Jan Bogaert, Amy M. Mitchell, Michael D. Flannery, Adrian D. Elliott, Chenglong Yu, Olivier Ghekiere, Tomas Robyns, Caroline M. Van De Heyning, Prashanthan Sanders, Jonathan M. Kalman, Monique Ohanian, Magdalena Soka, Emma Rath, Eleni Giannoulatou, Renee Johnson, Paul Lacaze, Lieven Herbots, Rik Willems, Diane Fatkin, Hein Heidbuchel, André La Gerche

**Affiliations:** Faculty of Medicine and Life Sciences, Limburg Clinical Research Center (LCRC), Hasselt University, Biomedical Research Institute, Diepenbeek, Belgium (G.C., O.G., L.H.).; Hartcentrum Hasselt (G.C., L.H.), KU Leuven, Belgium.; Department of Radiology (O.G.), KU Leuven, Belgium.; Jessa Ziekenhuis, Belgium. Department of Cardiovascular Sciences (G.C., R.D.B., M.C., P.C., T.R., R.W., A.L.G.), KU Leuven, Belgium.; Department of Movement Sciences (C.D.), KU Leuven, Belgium.; Department of Imaging and Pathology (J.B.), KU Leuven, Belgium.; Department of Cardiovascular Diseases (R.D.B., K.G., T.R., R.W.), University Hospitals Leuven, Belgium.; Department of Radiology (J.B.), University Hospitals Leuven, Belgium.; HEART (Heart Exercise and Research Trials) Lab, St Vincent’s Institute of Medical Research, Fitzroy, Australia (K.J., A.M.M., A.L.G.).; Exercise and Nutrition Research Program, The Mary MacKillop Institute for Health Research, Australian Catholic University, Melbourne Australia (K.J.).; Victor Chang Cardiac Research Institute, Darlinghurst, Australia (P.Y., M.O., M.S., E.R., E.G., R.J., D.F., A.L.G.).; Department of Medicine, University of Melbourne, Parkville, Australia (M.D.F., J.M.K., A.L.G.).; Centre for Heart Rhythm Disorders, University of Adelaide and Royal Adelaide Hospital, Australia (A.D.E., P.S.).; Department of Epidemiology and Preventive Medicine, School of Public Health and Preventive Medicine, Monash University, Melbourne, Australia (C.Y., P.L.).; Department of Cardiovascular Sciences, University of Antwerp, Belgium (C.M.V.D.H., H.H.).; Department of Cardiology, University Hospital Antwerp, Belgium (C.M.V.D.H., H.H.).; Department of Cardiology, Royal Melbourne Hospital, Australia (J.M.K.).; School of Clinical Medicine, Faculty of Medicine and Health, UNSW Sydney, Kensington, Australia (R.J., D.F.).; Cardiology Department, St Vincent’s Hospital, Darlinghurst, Australia (D.F.).; Cardiology Department, St Vincent’s Hospital Melbourne, Fitzroy, Australia (A.L.G.).; National Centre for Sports Cardiology, Fitzroy, Australia (A.L.G.).

**Keywords:** arrhythmias, cardiac, cardiomegaly, cardiomegaly, exercise-induced, cardiomyopathies, fibrosis, genetics, genome

## Abstract

**BACKGROUND:**

Exercise-induced cardiac remodeling can be profound, resulting in clinical overlap with dilated cardiomyopathy, yet the significance of reduced ejection fraction (EF) in athletes is unclear. The aim is to assess the prevalence, clinical consequences, and genetic predisposition of reduced EF in athletes.

**METHODS:**

Young endurance athletes were recruited from elite training programs and underwent comprehensive cardiac phenotyping and genetic testing. Those with reduced EF using cardiac magnetic resonance imaging (defined as left ventricular EF <50%, or right ventricular EF <45%, or both) were compared with athletes with normal EF. A validated polygenic risk score for indexed left ventricular end-systolic volume (LVESVi-PRS), previously associated with dilated cardiomyopathy, was assessed. Clinical events were recorded over a mean of 4.4 years.

**RESULTS:**

Of the 281 elite endurance athletes (22±8 years, 79.7% male) undergoing comprehensive assessment, 44 of 281 (15.7%) had reduced left ventricular EF (N=12; 4.3%), right ventricular EF (N=14; 5.0%), or both (N=18; 6.4%). Reduced EF was associated with a higher burden of ventricular premature beats (13.6% versus 3.8% with >100 ventricular premature beats/24 h; *P*=0.008) and lower left ventricular global longitudinal strain (–17%±2% versus –19%±2%; *P*<0.001). Athletes with reduced EF had a higher mean LVESVi-PRS (0.57±0.13 versus 0.51±0.14; *P*=0.009) with athletes in the top decile of LVESVi-PRS having an 11-fold increase in the likelihood of reduced EF compared with those in the bottom decile (*P*=0.034). Male sex and higher LVESVi-PRS were the only significant predictors of reduced EF in a multivariate analysis that included age and fitness. During follow-up, no athletes developed symptomatic heart failure or arrhythmias. Two athletes died, 1 from trauma and 1 from sudden cardiac death, the latter having a reduced right ventricular EF and a LVESVi-PRS >95%.

**CONCLUSIONS:**

Reduced EF occurs in approximately 1 in 6 elite endurance athletes and is related to genetic predisposition in addition to exercise training. Genetic and imaging markers may help identify endurance athletes in whom scrutiny about long-term clinical outcomes may be appropriate.

**REGISTRATION:**

URL: https://www.anzctr.org.au/Trial/Registration/TrialReview.aspx?id=374976&isReview=true; Unique identifier: ACTRN12618000716268.

**Clinical Perspective**

**What Is New?**

- Abnormal measures of cardiac function are not uncommon in elite endurance athletes, with 1 in 6 being found to have a reduction in left or right ventricular ejection fraction.
- Reduced ejection fraction was associated with increased ventricular ectopy and reduced global longitudinal strain, but fitness was preserved, and there was no excess in myocardial fibrosis.
- Both genetic susceptibility and environmental effects may contribute to ventricular remodeling in athletes.

**What Are the Clinical Implications?**

- The finding of reduced ejection fraction in the clinical evaluation of an elite athlete may be considered part of the athletic phenotypic spectrum rather than a marker of cardiac disease.
- These data provide cautious reassurance to inform current screening protocols (mandated by several sporting governing bodies) that do not currently provide guidance on the management of athletes with reduced ejection fraction.
- Asymptomatic athletes with reduced ejection fraction should be allowed to continue competitive sport, but continued follow-up is recommended.
- The risk of future dilated cardiomyopathy development in athletes with high polygenic risk score for indexed left ventricular end-systolic volume is currently unknown, and prospective follow-up is warranted.

**Editorial, see p 1416**

Cardiac adaptations resulting from habitual intense endurance exercise, often termed “the athlete’s heart,” include dilation of all cardiac chambers and a tendency to lower measures of systolic function.^1,2^ Endurance athletes with an abnormal ejection fraction (EF) present a clinically challenging overlap between athlete’s heart and dilated cardiomyopathy (DCM), as highlighted by Abergel et al, who documented reduced EF in 7% of cyclists who competed in the 1995 and 1998 Tour de France.^1^ Also, intense endurance exercise has been linked to an increased prevalence of fibrosis and both atrial and ventricular arrhythmias,^3^ some of which might predispose to sudden cardiac death.^4^

It is commonly assumed that cardiac remodeling in athletes is induced by the mechanical stress of sustained exercise. However, it is possible that variability in cardiac dilation and EF in athletes exposed to similar exercise burdens may be explained in part by differences in genetic predisposition. In virtually all nonathletic clinical settings, reduced EF is associated with an increase in adverse cardiovascular events. It has been assumed that ostensibly healthy athletes are an exception, but this hypothesis has not been interrogated. With increased uptake of preparticipation cardiovascular screening, asymptomatic ventricular dysfunction is a prevalent finding, and yet there is scant evidence to guide evaluation and management.

Therefore, we aimed to study the prevalence, causes, and consequences of reduced left or right ventricular (RV) EF in a prospective cohort of healthy young elite endurance athletes.

## METHODS

The data that support the findings of this study are available from the corresponding author upon reasonable request.

### Study Population

The study cohort consisted of elite competitive endurance athletes from the Pro@Heart (Prospective Athletic Heart) and ProAFHeart (Prospective Atrial Fibrillation Athletic Heart) studies. These international multicenter prospective trials aimed to investigate the effect of training load and genotype on the variability of structural, functional, and electrical exercise-induced cardiac remodeling in elite competitive endurance athletes, and the full study protocol has been detailed.^5^ Athletes were recruited through their sports federation or team, who were made aware of the study through advertisements, media, and scientific presentations. The athletes were enrolled to undergo the study investigations at 1 of 5 medical research facilities: (1) Baker Heart and Diabetes Institute, Melbourne, Australia; (2) Royal Adelaide Hospital, Adelaide, Australia; (3) Gasthuisberg University Hospital, Leuven, Belgium; (4) Jessa Ziekenhuis, Hasselt, Belgium; and (5) Universitair Ziekenhuis Antwerpen, Antwerp, Belgium. Athletes were eligible if they (1) competed in an endurance sport in which aerobic conditioning is a principal component of performance (eg, triathlon, cycling, rowing/canoeing, cross-country skiing, distance running ≥1500 meters and swimming ≥400 meters), and (2) competed at a national or international level. Athletes with a known preexisting cardiac or respiratory condition or with contraindications to cardiac magnetic resonance imaging (CMR) were excluded. Athletes all self-reported having European ancestry.

The study is listed in the Australia New Zealand Clinical Trials Registry (URL: https://www.anzctr.org.au/Trial/Registration/TrialReview.aspx?id=374976&isReview=true. Unique identifier: ACTRN12618000716268).

Two comparison groups were used for the genetics analyses: (1) a cohort of probands with familial DCM recruited for genetics research at the Victor Chang Cardiac Research Institute and (2) a control group of healthy older individuals with no history of diagnosed cardiovascular disease events enrolled in the ASPREE trial (Aspirin in Reducing Events in the Elderly; NCT01038583).^6^ We included in this study DCM probands and ASPREE participants with non-Finnish European ancestry.

Protocols were approved by the Human Research Ethics Committees at each of the recruiting sites in Australia and Belgium, and all participants provided written consent.

### CMR Imaging

CMR was performed using a 1.5T or 3.0T magnetic resonance imaging scanner (Magnetom Aera 1.5T, Prisma 3.0T, or Skyra 3.0T, Siemens Healthineers, Erlangen, Germany; Ingenia, Achieva or Ambition 1.5T, Philips Medical Systems, Best, The Netherlands). A steady-state free precession dynamic echo-gradient sequence was used to obtain cine-loops during breath-hold in short axis and 4-chamber views. Left ventricular (LV) mass (not including papillary muscles and trabeculae) and biventricular volumes and function were quantified by experienced investigators using customized analysis software (RightVol, Leuven, Belgium) and then were normalized to body surface area to derive indexed LV and RV end-diastolic and end-systolic volumes and LV mass. A reduced EF was defined as LVEF <50% or RVEF <45% on CMR. Myocardial fibrosis was assessed by late gadolinium enhancement (LGE) imaging on breath hold phase-sensitive inversion recovery sequences 10 minutes after administration of gadolinium-diethylenetriamine penta-acetic acid. Athletes evaluated at the University Hospitals Leuven and Baker Heart and Diabetes Institute Melbourne underwent CMR during exercise on a supine ergometer at 25%, 50%, and 66% of maximal power determined by previous upright exercise testing. Increases of EF by <11% from rest to peak exercise were defined as abnormal LV and RV contractile reserve, respectively.^7,8^

### Echocardiography

Two-dimensional transthoracic echocardiography was performed (Vivid E9 or E95 ultrasound system (GE Healthcare, Horton, Norway) to assess LV global longitudinal strain (GLS) and diastolic function using established Doppler parameters (peak E and A wave velocities, E/A ratio, tricuspid regurgitation flow velocity) and tissue Doppler parameters (septal, lateral, and averaged E’, E/E’).

Analysis of all CMR and echocardiographic images were performed at 1 of 2 core laboratory facilities, both of which used the same software and methods—Baker Heart and Diabetes Institute for the Australian Centers and Gasthuisberg University Hospital for the Belgian Centers. A central image repository was created for the assessment of variability between core center analyses.

### ECG and Holter Monitoring

A resting 12-lead ECG and 24-hour Holter monitoring were performed to determine heart rate boundaries and to detect arrhythmias. Rhythm monitoring was performed using a Spiderview Holter device (Microport, Clamart, France) and analyzed offline using SyneScope software (ELA Medical, Paris, France) at the Belgian centers and using a PocketECG Holter (MEDICalgorithmics, Warsaw Poland) at the Australian centers. Bradycardia was defined as a heart rate <50/min. A cardiac pause was defined as an interruption in the ventricular rate >2 s and nonsustained ventricular tachycardia as >3 consecutive ventricular beats at >100/min and lasting <30 s. An increased burden of atrial premature beats and ventricular premature beats (VPBs) was defined as >100/24 h and >500/24 h.

### Exercise Stress Testing

Maximal oxygen consumption was determined using a sports-specific ergometer when possible. Respiratory gas exchange was analyzed using a breath-by-breath open circuit spirometry system. Maximal oxygen consumption was determined as the highest 30-s average oxygen consumption.

### Genetic Analyses

Peripheral blood samples were collected, and DNA was extracted following standard protocols.^9^ Genotyping was performed using the Axiom Precision Medicine Diversity Array (v2.0; Thermo Fisher Scientific, CA) following standard protocols as described previously.^10^ Array quality control was performed following the best practices workflow according to the manufacturer’s instructions with in-house pipelines. Thresholds of >82% for dish quality control and >97% call rate were applied to each sample. Variant imputation was performed on the Michigan imputation server running minmac4 with the Haplotype Reference Consortium.^11^ Genomic risk analysis was performed with plink (v1.9) using both the genotyped and imputed variant calls with hg19 (GRCh37) coordinates. A polygenic risk score for indexed LV end-systolic volume that had been previously associated with incident DCM in the general population (LVESVi-PRS; PRS000666), and a polygenic risk score for hypertrophic cardiomyopathy (HCM) (HCM-PRS; PGS000778) were derived.^12,13^ A total of 20 of 28 variants were used for LVESVi-PRS with all 20 variants used for the HCM-PRS. Sequencing analysis to identify pathogenic rare variants in cardiomyopathy-associated genes was considered outside the scope of this study.

### Statistical Analysis

Data were analyzed using SPSS Statistics version 27 (IBM Corporation, Armonk, NY). Normality was tested using the Shapiro-Wilk test. Continuous variables are presented as means (±SDs) or as medians (with 25% and 75% percentiles) accordingly and categorical variables as proportions. Athletes with an isolated reduced LVEF, isolated reduced RVEF, and reduced LVEF and RVEF were combined in a single group (“reduced EF”) to increase statistical power. Between-group differences in continuous variables were assessed using ANOVA, unpaired *t* test, or Mann-Whitney test as appropriate. A chi-square test was used for dichotomous variables. The cardiac response to exercise was assessed using a mixed linear model with subject group, exercise stage, and their interaction as fixed effects. Univariate and multivariate logistic regression was performed to assess the determinants of a reduced EF. A 2-tailed *P* value <0.05 was considered statistically significant.

## RESULTS

### Clinical Characteristics

**Figure 1.**
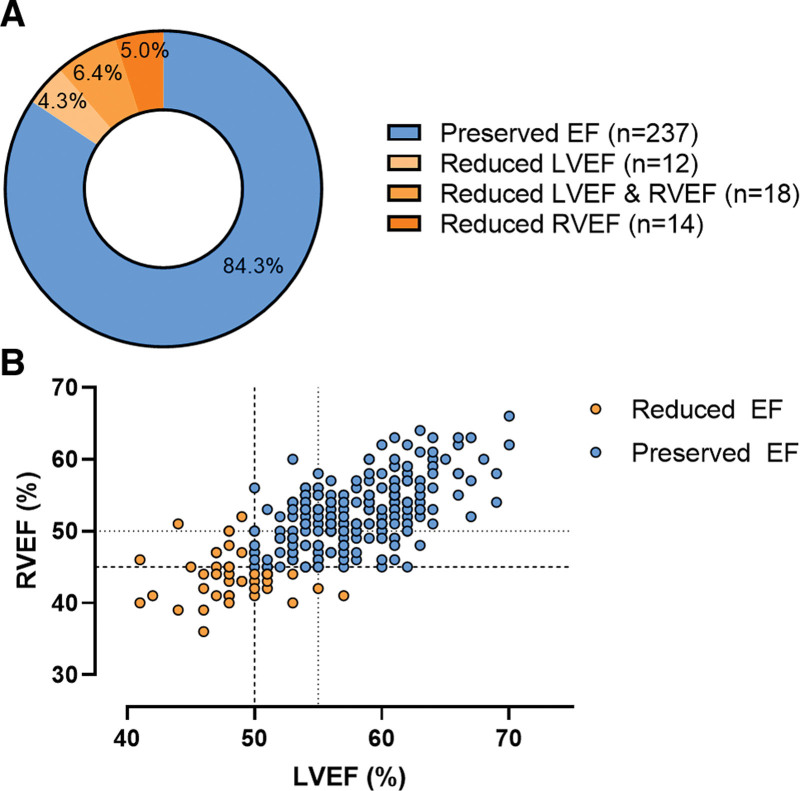
**Population distribution on the basis of ejection fraction and the relationship between LVEF and RVEF. A**, Doughnut chart of the population distribution on the basis of ejection fraction. A reduced EF is defined as an LVEF <50% or an RVEF <45%. **B**, The relationship between LVEF and RVEF in endurance athletes. The dashed vertical and horizontal lines represent the cutoffs for a reduced LVEF (50%) and RVEF (45%), respectively. The dotted vertical and horizontal lines represent the cutoffs for a borderline LVEF (55%) and RVEF (50%). EF indicates ejection fraction; LV, left ventricular; and RV, right ventricular.

A total of 281 elite endurance athletes were investigated. A total of 44 (15.7%) had reduced EF on CMR: isolated reduced LVEF <50% (n=12), isolated reduced RVEF <45% (n=14), or reduced LVEF + RVEF (n=18). The population distribution of LVEF and RVEF is shown in Figure 1A. The majority of athletes with reduced LVEF also had a tendency to have reduced RVEF (Figure 1B), thereby indicating biventricular involvement in most cases. Age, sex, body mass index, blood pressure, sports type, and training load were similar between athletes with and without reduced EF (Table 1). Both groups had similarly high levels of exercise capacity (maximal oxygen consumption, 63.5 [58.1–68.6] versus 62.3 [55.7–66.8] mL/min/kg; *P*=0.113).

**Table 1.**
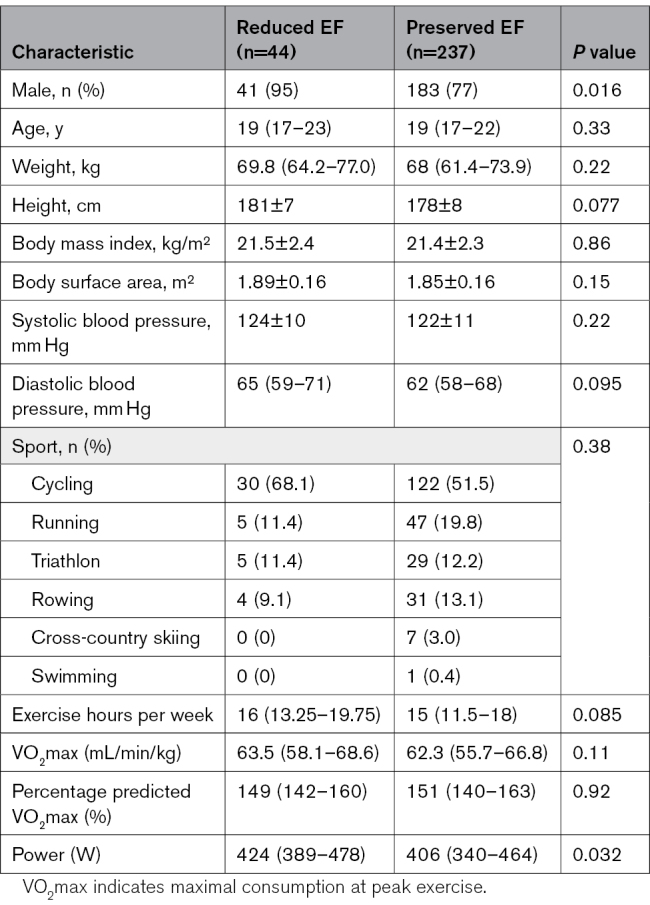
Baseline Characteristics of Elite Endurance Athletes With Reduced Ejection Fraction and Preserved Ejection Fraction

### Resting Assessment of Cardiac Structure and Function

**Figure 2.**
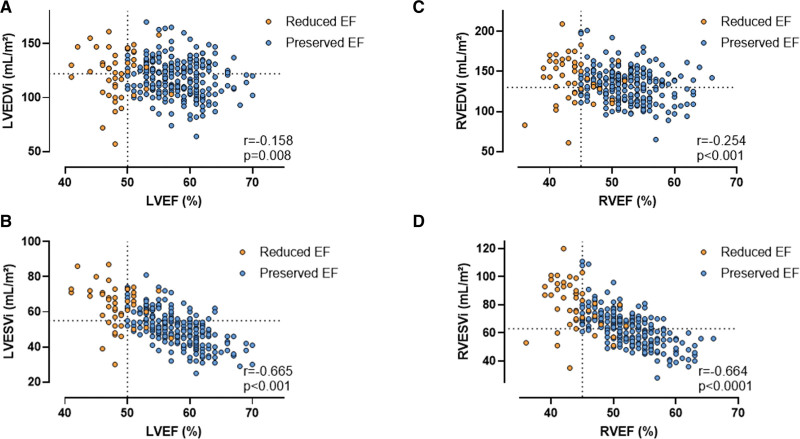
**The correlation between ejection fraction, end-diastolic volumes, and end-systolic volumes.** Scatter plot and correlation analysis between (**A**) LVEF and LVEDVi, (**B**) LVEF and LVESVi, (**C**) RVEF and RVEDVi, and (**D**) RVEF and RVESVi. The vertical dotted lines represent the cutoff values of reduced LVEF at 50% and RVEF at 45%. The horizontal dotted lines represent the upper limit of the 99% CI of LVEDVi (122 mL/m²), LVESVi (55 mL/m²), RVEDVi (130 mL/m²), and RVESVi (63 mL/m²) as reported by D’Ascenzi et al.^14^ EF indicates ejection fraction; LV, left ventricular; LVEDVi, indexed left ventricular end-diastolic volume; LVESVi, indexed left ventricular end-systolic volume; RV, right ventricular; RVEDVi, indexed right ventricular end-diastolic volume; and RVESVi, indexed right ventricular end-systolic volume.

Table 2 shows the cardiac structure and function at rest. LV end-diastolic volume was comparable between groups (123±23 versus 118±17 mL/m²; *P*=0.150). However, athletes with reduced EF had higher RV end-diastolic volumes (143 [124–162] versus 133 [116–144] mL/m²; *P*=0.005). Athletes with reduced EF had higher end-systolic LV and RV volumes (LVESVi, 65 [54–72] versus 50 [43–56] mL/m²; RV end-systolic volume indexed, 82 [67–93] versus 63 [54–70] mL/m²; *P*<0.001). Consistent with these categorical differences, there were modest negative correlations between biventricular end-diastolic volumes and EF and strong negative correlations between biventricular end-systolic volumes and EF (Figure 2).

**Table 2.**
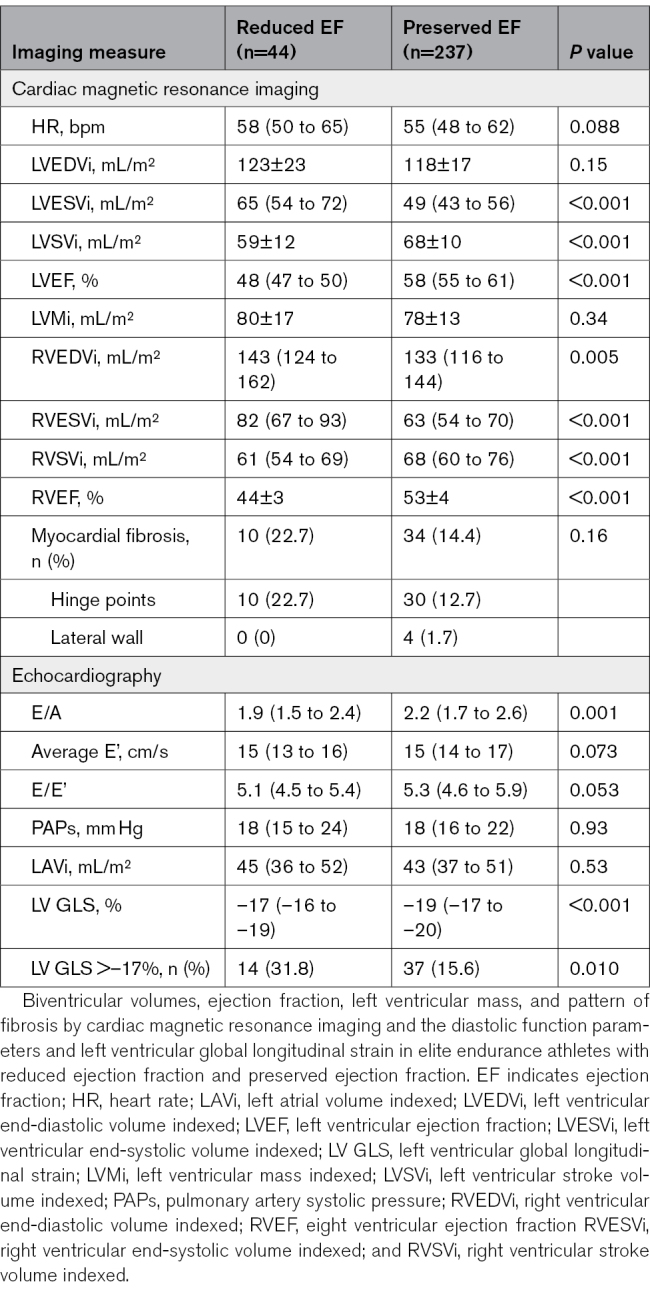
Cardiac Imaging Characteristics

**Figure 3.**
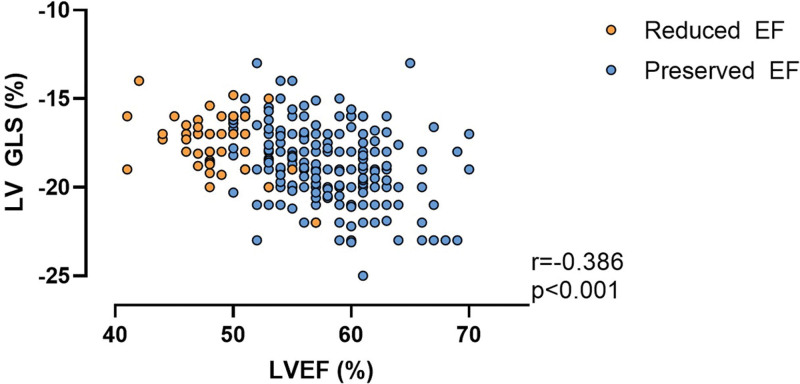
**Correlation between LVEF and LV GLS.** For athletes with reduced EF (orange) and preserved EF (blue). EF indicates ejection fraction; GLS, global longitudinal strain; and LV, left ventricular.

When compared with those with preserved EF, athletes with reduced EF had lower LV GLS (–17% [–16% to –18.7%] versus –19% [–17.2% to –20%]; *P*<0.001) and more frequently met criteria for reduced LV GLS (defined as >–17.0%; 31.8% versus 15.6%; *P*=0.010). Consistent with this, there was a moderate negative correlation such that lower EF was associated with reduced GLS (*r*=–0.386; *P*<0.001; Figure 3). LV diastolic function was similar between groups except for lower E/A ratio in athletes with reduced EF (1.9 [1.5–2.4] versus 2.2 [1.7–2.6]; *P*=0.001).

LGE, a marker of macroscopic myocardial fibrosis, was present in 44 (15.7%) athletes with no difference between groups: reduced EF (n=10 of 44, 22.7%), preserved EF (n=34 of 237, 14.4%; *P*=0.160). All 10 athletes with reduced EF had LGE located in the interventricular septum at the “hinge point” of RV attachment. Of the 34 LGE-positive athletes with a preserved EF, 30 had RV hinge point fibrosis, whereas 4 had LV mid/epicardial lateral wall fibrosis.

### Cardiac Function During Exercise

**Figure 4.**
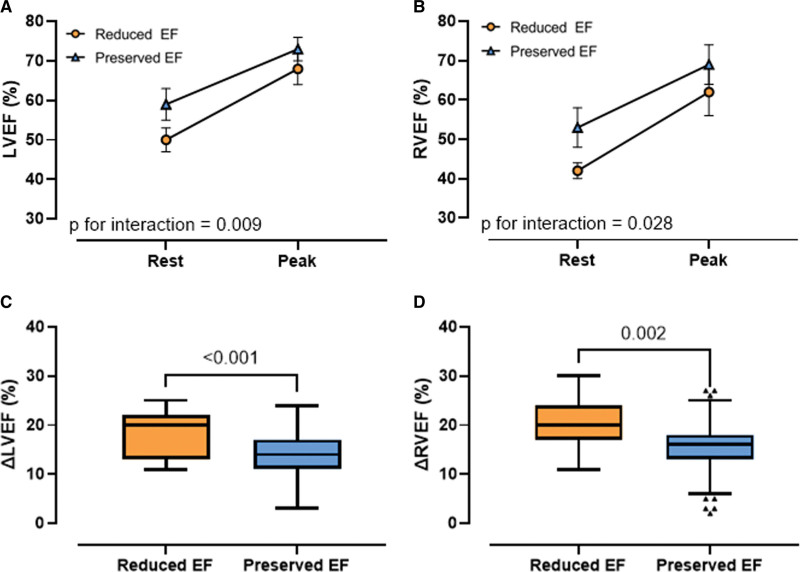
**Changes in ejection fraction during exercise.** Changes in LVEF (**A**) and RVEF (**B**) from rest to peak exercise in athletes with a reduced (orange dots) and preserved EF (blue triangles). Box-and-whiskers plots of delta LVEF (**C**) and delta RVEF (**D**) between rest and peak exercise in athletes with a reduced (orange) and preserved EF (blue). EF indicates ejection fraction; LV, left ventricular; and RV, right ventricular.

Acute responses to exercise were evaluated using an exercise CMR protocol. Heart rate and power output at peak exercise were similar between those with reduced and preserved EF. Although EF at peak exercise remained lower in athletes with reduced EF (LVEF, 68%±3% versus 73%±4%; RVEF, 62%±6% versus 69%±5%; *P*<0.001), there was a relatively greater overall increment in EF during exercise in athletes with reduced EF (LVEF, +18%±5% versus +14%±4%; *P*<0.001; RVEF, +19%±5% versus +15%±5%; *P*=0.002; Figure 4; Table S1).

### Arrhythmia Propensity

Resting 12-lead ECG properties are presented in Table S2. Athletes with reduced EF had marginally longer QRS duration compared with athletes with preserved EF (104±8 versus 99±9; *P*=0.001), but all other parameters were similar. On 24-hour Holter monitoring, athletes with reduced EF had a higher burden of VPBs (13.6% versus 3.8% >100 VPB/24 h; *P*=0.008; Table 3). Episodes of nonsustained ventricular tachycardia were seen in only 3 athletes, all of whom had preserved EF (0 versus 1.3%; *P*=0.45). Average, maximum, and minimum heart rates, pauses >2 s, and atrial premature beat were similar between groups.

**Table 3.**
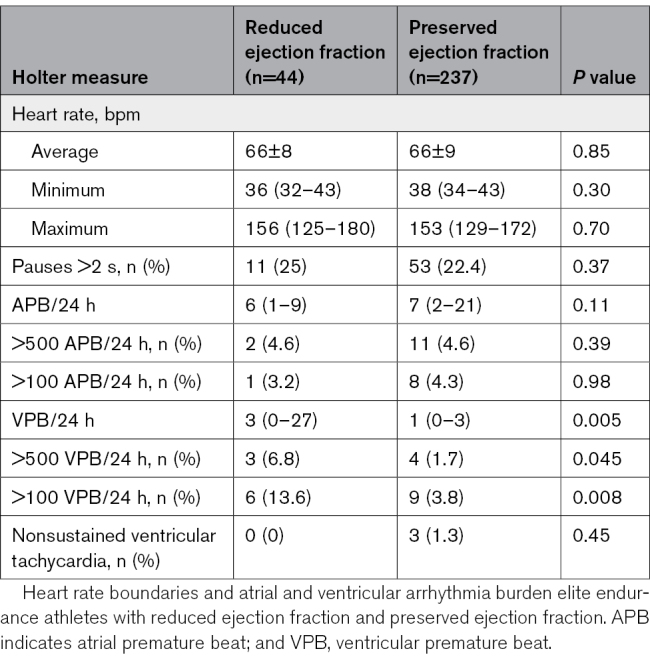
Results of 24-Hour Holter Monitoring

### Genetic Predisposition to Reduced EF

Genetic analysis was performed in 271 athletes (reduced EF, n=43; preserved EF, n=228; DNA unavailable, n=10). To determine whether there was a genetic contribution to reduced EF in endurance athletes, we evaluated the distribution of LVESVi-PRS within the athletic cohort using a PRS that has been associated with increased LVESVi and incident DCM. The mean LVESVi-PRS was significantly higher in athletes with reduced EF compared with athletes with preserved EF (0.57±0.13 versus 0.51±0.14; *P*=0.009). Athletes in the top quartile, quintile, and decile of LVESVi-PRS scores had a progressive increase in risk of reduced EF, being 11-fold higher for the top versus bottom deciles (Table 4).

**Table 4.**
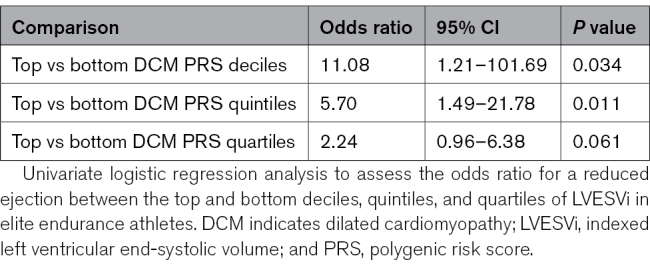
Univariate Logistic Regression for a Reduced Ejection Fraction by LVESVi-PRS

**Figure 5.**
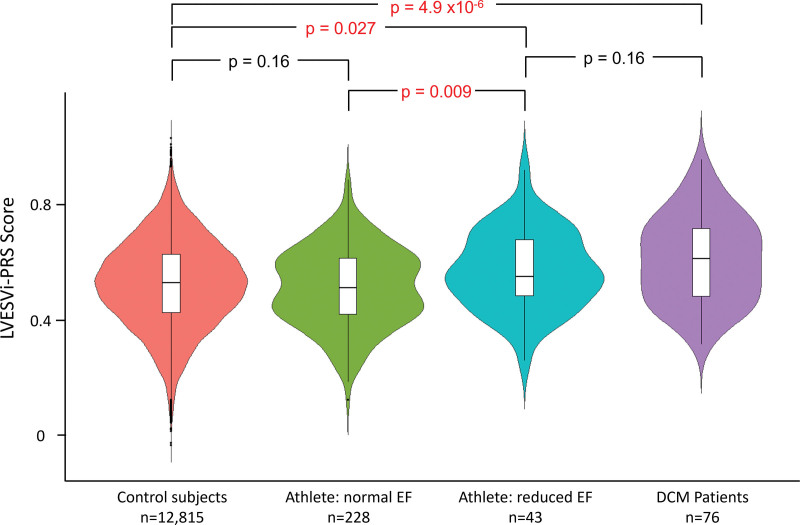
**Polygenic risk score for LVESVi: violin plots showing the distribution of LVESVi-PRS in athletes with a reduced or normal EF, in comparison with a healthy elderly population from the ASPREE study^6^ and patients with established DCM.** Mean LVESVi-PRS in athletes with reduced EF is higher than in control subjects and athletes with normal EF, but similar to patients with DCM. Data for mean ± interquartile range in each group are shown. ASPREE indicates Aspirin in Reducing Events in the Elderly; DCM, dilated cardiomyopathy; EF, ejection fraction; LVESVi, indexed left ventricular end-systolic volume; and PRS, polygenic risk score.

To determine how the range of LVESVi-PRS values in athletes with and without reduced EF correlated with diseased and healthy cohorts, we next compared mean LVESVi-PRS values in athletes with healthy control subjects from the ASPREE study (n=12 815) and affected probands with familial DCM (n=76). There were significant differences between these 4 groups (ANOVA, *P*=1.5×10^–6^). Of note, there were no significant differences in mean LVESVi-PRS between healthy controls and athletes with preserved EF. However, values in both groups were significantly lower than in athletes with reduced EF and patients with DCM (Figure 5).

In a multivariate analysis that included age and fitness, male sex and greater LVESVi-PRS were the only significant predictors of reduced EF (Table S3). An inverse relationship has previously been reported between PRS for DCM and HCM. Consistent with this, we found that mean HCM-PRS was significantly lower in athletes with reduced EF compared with those without reduced EF (1.07±0.26 versus 1.21±0.26; *P*=0.002).

### Follow-Up

Athletes were followed for a median of 4.4 (range, 0.2–6.9) years. No athletes developed symptomatic heart failure or documented sustained atrial or ventricular arrhythmias. Two athletes, both men with reduced EF, died suddenly during competition; 1 death was due to traumatic injury after an unprovoked accident while cycling and 1 was due to sudden cardiac death. The athlete with sudden cardiac death had reduced RVEF (RVEF=44%) and LVEF in the lower range of normal (LVEF=51%) at rest, a VPB count of 139/24 h, no evidence of LGE, and high genetic risk (LVESVi-PRS in the top 5%). A postmortem examination revealed microscopic evidence of patchy myocardial fibrosis of both ventricles, raising the possibility of a cardiomyopathic process. Testing of known cardiomyopathy-associated genes did not identify a potentially causative variant, and clinical evaluation of first-degree relatives has not identified any suspicion of cardiac disease. He had undergone regular testing for performance-enhancing drugs, had not experienced any recent illness, and had not previously been diagnosed with myocarditis.

## DISCUSSION

Athletes generally enjoy excellent health, but a minority present with extreme changes in cardiac structure and function that invoke consideration of underlying cardiac pathology. Combining comprehensive assessment of athletic conditioning, cardiac remodeling, and genomic analysis, we found that 1 in 6 elite endurance athletes has reduced EF, thereby raising the suspicion of subclinical DCM. Supporting this premise was an association with reduced myocardial strain, increased VPB, and an enrichment of background genetic variation similar to that seen in patients with DCM.^12^ On the other hand, athletes with reduced EF had similar cardiorespiratory fitness to those with preserved EF, enhanced augmentation of EF with exercise, similar low levels of myocardial fibrosis, and a good short-term clinical outcome. Thus, it could be argued that the extreme cardiac remodeling found in this group of highly trained young athletes should be regarded as healthy remodeling. However, the overlap in some clinical and genetic traits between more extreme athletic remodeling and DCM highlights the need for longer-term prospective follow-up, as is planned to continue in our Pro@Heart study.^5^

Using the gold standard of CMR, we found that reduced EF is relatively common, being present in nearly 16% of elite and highly trained endurance athletes. EF is a simple and imperfect measure of cardiac systolic function but has established prognostic significance. In a study of 4257 unselected individuals from the Framingham Study, asymptomatic LV dysfunction (LVEF <50%) was identified in 6.0% of men and 0.8% of women and was associated with a 3-fold increase in incident heart failure and a 1.6-fold increase in mortality risk.^15^ Similarly, in an analysis of 7749 people from 2 community-based studies, EF and increased LV mass were independently associated with sudden cardiac death risk.^16^ To our knowledge, there have not been any reports of populations in which a low EF has been associated with benign outcomes. By virtue of the superb physical capacity of endurance athletes, it has often been assumed that extreme measures of remodeling and function in this group are part of the broad physiological (ie, benign) remodeling. Our current data support this premise in some respects, but also raise the possibility of pathology in some instances.

To investigate the possibility of an underlying DCM (ie, nonbenign remodeling), we performed more in-depth evaluation of cardiac function using both myocardial deformation imaging and assessment of contractile reserve during exercise. GLS has been argued to be more sensitive than EF for identifying subclinical myocardial dysfunction.^17,18^ In keeping with the hypothesis of an underlying cardiomyopathy substrate, we found significant reduction of myocardial strain in athletes with reduced EF. We have previously promoted exercise imaging as a means of differentiating physiological from maladaptive remodeling in athletes.^7,19,20^ Thus, our finding that athletes with reduced EF had excellent exercise capacity and enhanced cardiac reserve (greater increase in EF during exercise) might provide reassurance.

Myocardial fibrosis is an established feature of maladaptive cardiac remodeling. Using CMR as the reference method to assess focal myocardial replacement fibrosis, 13% of all athletes in our study had evidence of myocardial fibrosis, but with no differences in prevalence between those with preserved EF or reduced EF. Myocardial fibrosis was mostly located at the RV insertion points to the interventricular septum, which has generally been considered a benign observation.^21–23^ Contrary to hinge-point fibrosis, subepicardial and midmyocardial fibrosis may be associated with potentially life-threatening arrhythmias.^24–26^ It is interesting that midmyocardial fibrosis of the LV lateral wall was seen in only 3 athletes, all of whom had preserved EF.

In contrast with these reassuring CMR findings pertaining to fibrosis and contractile reserve, athletes with reduced EF had a higher burden of VPBs (both when using cutoffs >100/24 h and >500/24 h). This excess VPB in reduced EF was substantially higher than that previously reported in young competitive athletes and healthy nonathletes.^27–29^ Frequent VPBs are a known risk marker for heart failure and mortality in the general population,^30^ but its significance in athletic populations remains unclear. We did not observe a higher prevalence of nonsustained ventricular tachycardia in athletes with reduced EF, although the cohort size precludes meaningful assessment of rare arrhythmias.

The causes of reduced EF in endurance athletes have not previously been investigated. Our data suggest, for the first time, that this is not entirely explained by exercise effects alone. Within the athletic cohort, we found significant differences in mean LVESVi-PRS between those with/without reduced EF, pointing to a genetic contribution to exercise-induced remodeling. The extent to which gene-environment interactions influence susceptibility to exercise effects or modify remodeling responses is currently unclear. Further prospective evaluation is needed to determine the natural history of high LVESVi-PRS in athletes and the risk of incident DCM. Values for athletes with reduced EF were not significantly different from those in a group of patients with familial DCM, suggesting a similar background genetic risk. However, whether exercise and lifestyle factors might mitigate against overt DCM development in athletes remains to be determined. Further studies are also needed to determine whether reduced EF in a subset of cases may in fact represent preclinical DCM caused by inherited rare genetic variants. Although our sample is small, we contend that the association between high LVESVi-PRS, reduced EF, increased VPB frequency, and the case of sudden cardiac death in an athlete with all of these features is sufficient to warrant further scientific scrutiny.

Last, there exists a further group of athletes with preserved EF but reduced LV GLS, reduced contractile reserve, nonsustained ventricular tachycardia, or high VPB burden. We contend that this represents a group that also warrants longer-term follow-up.

### Limitations

The current analysis was mainly performed on cross-sectional data, which limits conclusions on the prognosis of a reduced EF. The limited 4.4 years of follow-up in this cohort will be extended given that the Pro@Heart and ProAFHeart trials are long-term prospective trials.^5^ The unexpectedly high mortality rate in our cohort to date (0.7%) likely represents a Type I error, but it does emphasize the importance of adequately powered prospective studies to assess clinical end points in endurance athletes.

To achieve a sufficient sample size, the 3 subgroups of athletes with LV, RV, or biventricular reductions in EF were combined. This precludes investigations into cardiomyopathic subtypes such as DCM and arrhythmogenic cardiomyopathy. However, we identified a strong association between LVEF and RVEF that argued against a dominant subtype.

All athletes were White; thus, results may not be applicable to athletes from different ethnicities. We also had a greater proportion of male than female athletes.

There is potential for a selection bias in which athletes may have been motivated to enroll to assess mild symptoms or to appease concern of a family history of cardiovascular disease. This potential bias was limited given the young age of athletes and the fact that the questionnaire performed at inclusion confirmed the absence of symptoms and the negative family history of first-degree relatives.

### Conclusions

Reduced EF is a frequent finding in highly trained elite endurance athletes. Despite excellent cardiorespiratory fitness, enrichment of cardiomyopathy genetic traits and an increase in ventricular ectopy were observed among athletes with reduced EF. The long-term health impact in these athletes requires further scrutiny.

## ARTICLE INFORMATION

### Sources of Funding

This work was supported by the National Health and Medical Research Council of Australia (APP1130353), Baker-Royal Melbourne Hospital Seed Grant, Heart Foundation of Australia, Australian Cardiovascular Alliance/Bioplatforms Australia Research Catalyst Program, and the Perpetual Impact Philanthropy Program. The ASPREE study was supported by a Flagship cluster grant (including the Commonwealth Scientific and Industrial Research Organisation, Monash University, Menzies Research Institute, Australian National University, University of Melbourne) and grants (U01AG029824 and U19AG062682) from the National Institute on Aging and the National Cancer Institute at the National Institutes of Health, by grants (334047 and 1127060) from the National Health and Medical Research Council of Australia, and by Monash University and the Victorian Cancer Agency. Dr Lacaze is supported by a National Heart Foundation Future Leader Fellowship (102604). Dr Claessen is supported by the Belgian Heart Foundation 2021 Léon Dumont Prize.

### Disclosures

None.

### Supplemental Material

Tables S1–S3

## APPENDIX

Pro@Heart Consortium: Sofie Van Soest; Youri Bekhuis, MD; Rik Pauwels, MD; Jarne De Paepe, MD; Peter Hespel, MSc, PhD; Steven Dymarkowski, MD, PhD; Tom Dresselaers, PhD; Hielko Miljoen, MD, PhD; Kasper Favere, MD; Bernard Paelinck, MD, PhD; Dorien Vermeulen, MD; Isabel Witvrouwen, MD, PhD; Dominique Hansen, MSc, PhD; Bert Op’t Eijnde, MD; Daisy Thijs, BSc; Peter Vanvoorden, MD; Kristof Lefebvre, MD; Paolo D’Ambrosio, MBBS; Stephanie Rowe, MBBS; Elizabeth Paratz, MBBS, PhD; Maria J. Brosnan, MBBS, PhD; David L. Prior, MBBS, PhD, Department of Cardiovascular Sciences (S.V.S., Y.B., R.P., J.D.P.), Department of Movement Sciences (P.H.), Department of Imaging and Pathology (S.D.), KU Leuven, Belgium. Department of Cardiovascular Diseases (S.V.S., Y.B., R.P., J.D.P.), Department of Radiology (S.D., T.D.), University Hospitals Leuven, Belgium. Faculty of Medicine and Life Sciences, Limburg Clinical Research Center (LCRC), Hasselt University, Biomedical Research Institute, Diepenbeek, Belgium (Y.B., R.P., J.D.P., D.H.). Hartcentrum Hasselt (D.T., P.V.), Jessa Ziekenhuis, Belgium. Department of Cardiovascular Sciences, University of Antwerp, Belgium (H.M., K.F., B.P., D.V., I.W.). Department of Cardiology, University Hospital Antwerp, Belgium (H.M., K.F., B.P., D.V., I.W.). REVAL/BIOMED, Hasselt University, Diepenbeek, Belgium (D.H., B.O.E.). Department of Cardiology, Algemeen Ziekenhuis Nikolaas, Sint-Niklaas, Belgium (K.L.). Department of Medicine, University of Melbourne, Parkville, Australia (P.D., S.R., E.P., D.L.P.). HEART (Heart Exercise and Research Trials) Lab, St Vincent’s Institute of Medical Research, Fitzroy, Australia (P.D., S.R., E.P.). Cardiology Department, St Vincent’s Hospital Melbourne, Fitzroy, Australia (S.R., E.P., M.J.B., D.L.P.). National Centre for Sports Cardiology, Fitzroy, Australia (M.J.B., D.L.P.).

## Supplementary Material


