# Supplementary material for: Reduced Ejection Fraction in Elite Endurance Athletes: Clinical and Genetic Overlap With Dilated Cardiomyopathy
Source: Circulation. 2023 Dec 18;149(18):1405–15. doi: 10.1161/CIRCULATIONAHA.122.063777 (PMC11062611; doi:10.1161/CIRCULATIONAHA.122.063777)
Supplement: Supplementary file 1 [file cir-149-1405-s001.pdf]

SUPPLEMENTAL MATERIAL

TABLE CAPTIONS

Supplementary table 1 – Exercise cardiac magnetic resonance imaging characteristics

The biventricular volumes and ejection fraction in response to exercise on a supine cycling exercise cardiac magnetic resonance imaging scan in elite endurance athletes with a reduced ejection fraction and a preserved ejection fraction from the Pro@Heart and ProAFHeart trials

Supplementary Table 2 – ECG characteristics

The electrocardiography characteristics of elite endurance athletes with a reduced ejection fraction and a preserved ejection fraction from the Pro@Heart and ProAFHeart trials

Supplementary Table 3 - Univariate and multivariate association with a reduced EF

Univariate and multivariate logistitic regression analysis to assess the determinants of a reduced ejection fraction in elite endurance athletes from the Pro@Heart and ProAFHeart trials.

Supplementary table 1 – Exercise cardiac magnetic resonance imaging characteristics

	Reduced EF (n=14)	Preserved EF (n=131)	p-value
Rest			
HR (bpm)	57 ± 9	55 ± 8	0.475
LVEDVi (mL/m²)	113 ± 29	114 ± 18	0.913
LVESVi (mL/m²)	53 (46 – 71)	47 (40 – 52)	0.014
LVSVi (mL/m²)	56 ± 14	67 ± 11	0.001
LVEF (%)	50 ± 3	59 ± 4	<0.001
RVEDVi (mL/m²)	133 (110 – 165)	126 (111 – 140)	0.235
RVESVi (mL/m²)	77 (62 – 97)	60 (51 – 67)	0.001
RVSVi (mL/m²)	61 (50 – 70)	65 (59 – 75)	0.037
RVEF (%)	43 ± 3	54 ± 5	<0.001
Average CI (L/min/m²)	3.3 (2.4 – 3.7)	3.5 (3.1 – 4.1)	0.091
Peak exercise			
HR (bpm)	151 (147 – 157)	151 (143 – 156)	0.343
Power (W)	195 (161 – 246)	186 (150 – 229)	0.455
LVEDVi (mL/m²)	111 ± 25	110 ± 17	0.841
LVESVi (mL/m²)	37 (29 – 44)	29 (24 – 35)	0.022
LVSVi (mL/m²)	75 ± 17	80 ± 12	0.213
LVEF (%)	68 ± 3	73 ± 4	<0.001
RVEDVi (mL/m²)	133 (108 – 160)	119 (105 – 132)	0.115
RVESVi (mL/m²)	51 (35 – 63)	37 (29 – 45)	0.005
RVSVi (mL/m²)	79 ± 18	82 ± 13	0.486
RVEF (%)	62 ± 6	69 ± 5	<0.001
Average CI (L/min/m²)	11.4 (10.1 – 14.3)	12 (10.5 – 13.4)	0.761
Delta (Peak – Rest)			
ΔHR (bpm)	95 (90 –103)	94 (88 – 103)	0.825
ΔLVSVi (mL/m²)	19 (15 – 24)	13 (9 – 17)	0.001
ΔLVEF (%)	18 ± 5	14 ± 4	0.001
ΔLVEF <11% - n (%)	1 (7.1)	22 (16.8)	0.699
ΔRVSVi (mL/m²)	22 ± 6	15 ± 8	0.001
ΔRVEF (%)	19 ± 5	15 ± 5	0.005
ΔRVEF <11% - n (%)	1 (7.1)	21 (16)	0.695
ΔAverage CI (L/min/m²)	8.0 (7.1 – 10.7)	8.3 (7.2 – 9.6)	0.731

HR: Heart rate - LVEDVi: Left ventricular end-diastolic volume indexed – LVESVi: Left ventricular end-systolic volume indexed – LVSVi: Left ventricular stroke volume indexed – LVEF: Left ventricular ejection fraction – LVMi: Left ventricular mass indexed – RVEDVi: Right ventricular end-diastolic volume indexed – RVESVi: Right ventricular end-systolic volume indexed – RVSVi: Right ventricular stroke volume indexed – RVEF: Right ventricular ejection fraction – CI: Cardiac index

Supplementary Table 2 – ECG characteristics

	Reduced EF (n=44)	Preserved EF (n=237)	p-value
HR (bpm)	55 (47 – 60)	52 (45 – 60)	0.438
PR (ms)	158 (137 – 174)	158 (141 – 180)	0.434
1st degree HB – n (%)	0 (0)	20 (8.4)	0.052
Axis – n (%)<br>Normal<br>Right<br>Left	 30 (68.2)<br>14 (31.8)<br>0	 178 (75.1)<br>54 (22.8)<br>5 (2.1)	0.293
QRS (ms)	104 ± 8	99 ± 9	0.001
BBB – n (%)<br>No<br>Incomplete right<br>Complete right<br>Left	 25 (56.8)<br>18 (41.9)<br>1 (2.3)<br>0 (0)	 155 (65.4)<br>78 (32.9)<br>4 (1.7)<br>0 (0)	0.551
TWI – n (%)	6 (13.6)	23 (9.7)	0.431
QTc (ms)	394 (378 – 409)	398 (384 – 415)	0.139

HR: Heart rate, HB: heart block, BBB: bundle branch block, TWI: T-wave inversion

Supplementary Table 3 - Univariate and multivariate association with a reduced EF

	**Univariate logistic regression**	**Multivariate logistic regression**
Variable	OR (95% CI)	OR (95% CI)
Age – per 1 year increase	1.015 (0.979 – 1.052)	
Sex – male vs female	4.033 (1.201 – 13.536)	3.006 (0.601 – 15.032)
Weight – per 1kg increase	1.018 (0.987 – 1.051)	
Height – per 1cm increase	1.041 (0.995 – 1.089)	
BMI – per 1kg/m² increase	1.015 (0.883 – 1.167)	
Systolic BP – per 1mmHg increase	1.018 (0.989 – 1.048)	
Diastolic BP – per 1mmHg increase	1.035 (0.997 – 1.074)	
Exercise – per 1h/week increase	1.071 (0.996 – 1.150)	
VO2max – per 1mL/min/kg increase	1.037 (0.997 – 1.079)	
LVESVi-PRS – per 0.01 increase	1.028 (1.005 – 1.052)	1.036 (1.011 – 1.061)

OR: Odds-ratio – CI: Confidence Interval
